# Correction: Paradoxical response reversal of top-down modulation in cortical circuits with three interneuron types

**DOI:** 10.7554/eLife.40642

**Published:** 2018-08-02

**Authors:** Luis Carlos Garcia del Molino, Guangyu Robert Yang, Jorge F Mejias, Xiao-Jing Wang

Garcia del Molino LC, Yang GR, Mejias JF, Wang X-J. 2017. Paradoxical response reversal of top-down modulation in cortical circuits with three interneuron types. *eLife* **6**:e29742. doi: 10.7554/eLife.29742.

Published 19, December 2017

In the *Firing-rate-based population model* section (Materials and methods) we say that, in the baseline activity steady-state, the background currents needed to obtain the desired rates are 136.4, 238.8, 92.6 and 91.8 pA. These values are wrong because they correspond to a previous version of the model with a different connectivity matrix and therefore using them, the baseline activity steady-state cannot be reproduced. The correct values of the background current for the baseline activity steady-state that correspond to the connectivity matrix given in Table 1 should be 114.7, 233.6, 94.3 and 89.9 pA for pyramidal, PV, SST and VIP respectively.

We have therefore updated the sentence “For example, for the baseline activity steady-state the background currents needed to obtain the desired rates (1, 10, 3 and 2 Hz for pyramidal, PV, SST and VIP, respectively) are 136.4, 238.8, 92.6 and 91.8 pA.’ to now read “For example, for the baseline activity steady-state the background currents needed to obtain the desired rates (1, 10, 3 and 2 Hz for pyramidal, PV, SST and VIP, respectively) are 114.7, 233.6, 94.3 and 89.9 pA.’

We apologize for any confusion that may have occurred.

The article has been corrected accordingly.

Updated 13, March 2020

Due to a computational error, the values displayed in the matrices in figures 2c and 2d are not correct. The actual values are given in the figure joined. We apologize for any confusion that may have occurred.

The corrected Figure 2 is shown here for reference:

**Figure 1:**
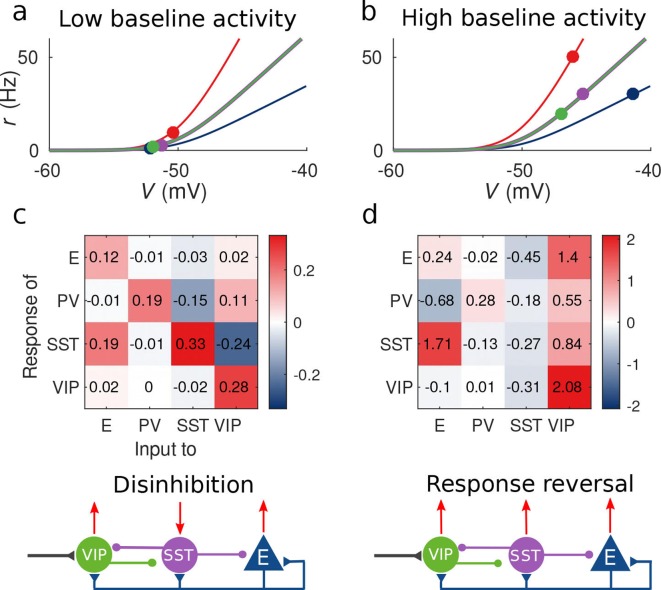


The original Figure 2 is shown here for reference:

**Figure 2:**
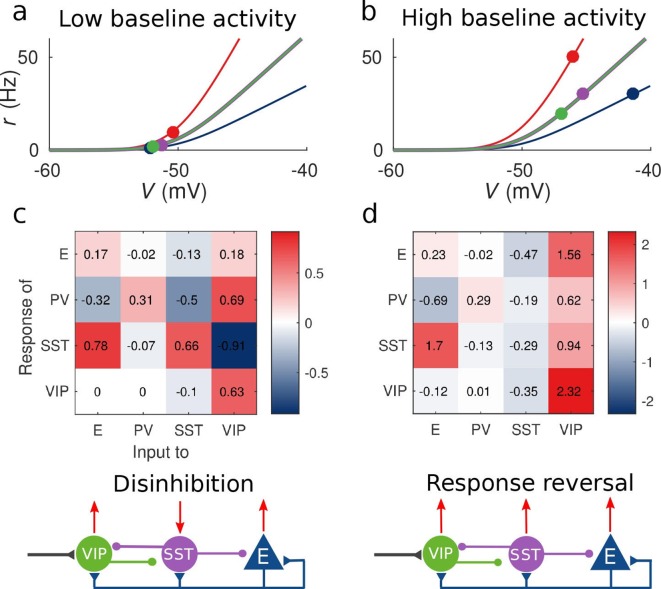


The article has been corrected accordingly.

